# Awareness of cardiovascular risk factors among university students in Turkey

**DOI:** 10.1017/S146342361900063X

**Published:** 2019-09-03

**Authors:** F. Esra Güneş, Nural Bekiroglu, Neşe Imeryuz, Mehmet Agirbasli

**Affiliations:** 1 Department of Biostatistics, Faculty of Medicine, Marmara University, İstanbul, Turkey; 2 Department of Gastroenterology, Faculty of Medicine, Marmara University, İstanbul, Turkey; 3 Department of Cardiology, Faculty of Medicine, Marmara University, İstanbul, Turkey

**Keywords:** awareness, cardiovascular diseases, healthy diet, risk factors, Turkey, young

## Abstract

**Aim:**

To determine the awareness of cardiovascular risk factors among university students in Turkey.

**Background:**

Cardiovascular disease (CVD) is the leading cause of death in developed countries. The use of tobacco products and unhealthy diet are prominent habits that increase the risk of CVD.

**Methods:**

Healthy university students (*n* = 2450) aged between 18 and 22 years in Istanbul filled out the questionnaire about the awareness of CVD risk factors and participated in this cross-sectional study. They were asked several questions with regard to the importance of CVD risk factors.

**Findings:**

The leading responses for men and women were, respectively, high cholesterol (58.3; 72.3%), stress (58.8; 71.8%), hypertension (50; 64.2%), smoking (53.1; 58.7%), obesity (46.8; 64.3%), diabetes (41.7; 52.7%), inactivity (43.3; 47.8%), and CVD in family history (31.8; 44.4%). Unhealthy diet (9.7; 15.3%), exposure to second-hand cigarette smoking (24.4; 34%), and poor socioeconomic status (22.6; 22.3%) were also considered to be important. The study also revealed that men disregard the risk factors more frequently. Another comparison between body mass index groups revealed that obese subjects gave significantly lower importance to cardiovascular risk factors.

**Conclusion:**

Observations indicate that awareness levels of CVD risk factors have to be improved among university students. It is emphasized that primary healthcare workers are very important in the screening of CVD risk factors in an opportunistic and systematic way and in providing consultancy on changing risky behaviors (diet, smoking, etc.). Therefore, it is of utmost importance that primary healthcare workers make interventions to reduce the risk level by determining the CVD risk.

## Background

Cardiovascular diseases (CVD) are one of the leading causes of death all over the world, and by 2030, more than 23 million people are expected to die from CVD according to the World Health Organization (WHO, 2013). A previous study in Turkish adult population (aged >20 years) indicated that the prevalence of heart diseases and CVD is 6.7% (6.2% in males and 7.3% in females) and 3.8% (4.1% in males and 3.5% in females), respectively (MedCHAMPS, 2012). Another study conducted in 2006 indicated the prevalence of CVD at 3%, 11%, and 27% in 39–49, 50–59, and >60 years of age, respectively (Onat *et al*., 2007; MedCHAMPS, 2012). According to the study by TUIK (2016), the leading mortality is from CVD, with 40.4% of deaths from noninfectious diseases.

The Framingham study has investigated the causes and formation of CVD in the United States since 1930s. According to the results of this study, the number of standard risk factors of CVD are elevated total cholesterol, high systolic blood pressure, cigarette smoking, low high-density lipoprotein cholesterol, obesity, unhealthy diet, poor nutritional status, age, family history, and male gender (D’Agostino *et al*., 2001).

In addition, CVD risk factors may depend on local and ethnic characteristics. For example, in sub-Saharan Africa, high blood pressure, high cholesterol, tobacco and alcohol use, and low fruit and vegetable consumption are significant risk factors for CVD (WHO, 2004). Prolonged exposure to these factors, especially in childhood, may cause CVD in the future (Leeder et al., 2004). TEKHARF (Onat *et al*., 2015), METSAR (Kozan *et al*., 2007) and Turkey Nutrition and Health Survey-2010 (2014) are epidemiological studies about CVD conducted in Turkey. Although this study overestimates some risk factors contributing for today’s populations and may not transfer well to ethnic groups properly, it gives an idea about risk factors of CVD. The perception of individuals regarding CVD not only determines their reactions to the diseases, but also affects the chance of preventive interventions against risks. Perception about the severity and risks of diseases has formed the basis of many health behavior theories (Ajzen et al., 1991; Weinstein et al., 1998; Molarius et al., 1999; Leventhal *et al*., 2003; Aljefree and Ahmed, 2015). While determining the steps in enhancing awareness about health, we should assess the level of awareness of the young and educated individuals, that is, university students. Baseline awareness is quite important in terms of planning effective interventions. Previous studies have demonstrated that understanding the severity of CVD is significantly lower compared with perception about cancer in the population (Mosca *et al*., 2004; Wang *et al*., 2009). Moreover, some studies indicated significant difference between the genders in terms of level of awareness (Aalto *et al*., 2007; Wang *et al*., 2009). Awareness is a prerequisite for the successful control of diseases and generates self-administered behavioral modification for diabetes, hypertension, and hypercholesterolemia (Langellier et al., 2012).

On the contrary, awareness of risk factors and healthy lifestyle reduces the risk of CVD, while it also reduces the growing CVD-related expenses. For instance, in 2004, nearly $368 billion was spent for CVD treatment in the United States (American Heart Association, 2003). In 2016, the economic burden of CVD in Turkey, including direct healthcare costs and indirect costs from lost productivity, was estimated at US$10.2 billion. Most of this burden ($5.9 billion) was associated with ischemic disease, while cerebrovascular disease costs were estimated at $4.2 billion (Balbay *et al*., 2018). Meanwhile, it is known that the savings in medical costs will increase economic productivity and quality of life (Thomas, 2005). The evaluation of population in terms of potential risks and preventive measures within the frame of social behavioral models can potentially increase the success level in the battle against CVD (Marteau and Weinman, 2006).

Thus, the aim of the present study was to determine the level of awareness among university students, especially freshman, in terms of risk factors such as obesity and unhealthy diet. Early adoption of such risky habits among young people may cause CVD in their future life.

## Methods

### Participants and data collection

Freshman university students (*n* = 2450) between 18 and 22 years enrolled to this cross-sectional study. Of the total participants, 1196 were boys (48.8%) and 1254 were girls (51.2%). The subjects were attending their first-year classes at health science schools in Istanbul, Turkey: a state university (Marmara University, *n* = 721) and a private university (Yeditepe University, *n* = 1804). All freshman students who were attending the university on the survey day and accepted the invitation to respond voluntarily to the questionnaire were included in the study. The response rate was quite high: out of 2525 students, 2450 (97.03%) answered the whole questionnaire.

All subjects were asked to complete the questionnaire; the questionnaires were completed in a classroom before an exam, when almost all of the students (so the coverage was 95%) were present.

Height and weight were self-reported by the subjects. Body mass index (BMI) values of participants were evaluated by dividing into categories: ⩽18.9, 19.0–24.9, 25.0–29.9, and ⩾30.0 kg/m^2^ was considered ‘underweight,’ ‘normal,’ ‘overweight,’ and ‘obese,’ respectively (Najjar and Rowland, 1987; Manson *et al*., 1995).

### The questionnaire

A committee consisting of a cardiologist, a gastroenterologist, a biostatistician, and a dietitian worked together to determine the content validity of the questionnaire. Before the application of the survey, a pilot study was performed. An initial draft of the questionnaire was given to 15 students of the same age and gender of the target population to test its feasibility. Necessary revisions were made on the initial draft.

The questionnaire used in the study was composed of 13 questions, which included demographic characteristics such as gender, age, smoking habits, family history of CVD of the participants, and indicated the level of knowledge and perception of CVD risk factors (obesity, stress, hypertension, drinking, smoking, family history of CVD, number of meals, diabetes, hypercholesterolemia, etc.), as well as necessary measures (smoking cessation, stress management, tension control, healthy diet, etc.). CVD risk factors in formation and measures to be taken were selected as specified in the literature (D’Agostino et al., 2001). The level of awareness was evaluated via five-point Likert-type questions. The questionnaire was completed by the participants.

The study was approved by the Institutional Review Board of Marmara University.

### Statistical analysis

Statistical analysis was performed using the Statistical Package for the Social Sciences software program (SPSS Inc., Chicago, IL, USA), version 15.0. Demographic data were analyzed by descriptive statistics, and data about the level of awareness were analyzed by chi-square statistics. Unpaired *t*-test was used for between-group comparisons of continuous variables, and *P*-value < 0.05 was considered significant.

## Findings

Of the 2450 subjects enrolled in the study, there were 1196 boys and 1254 girls. Mean height, weight, and BMI of the subjects were 171.99 ± 10.10 cm, 64.27 ± 13.62 Kg, and 21.57 ± 3.36 kg/m^2^, respectively. Demographic data of the subjects according to gender are demonstrated in Table 1. Accordingly, there were significant differences between boys and girls in terms of height, weight, and BMI with higher values in boys (*P* < 0.001 for all).

**Table 1.** Demographic characteristics of subjects

	Boys (*n* = 1196)		Girls (*n* = 1254)	
	Mean	SD	Mean	SD
Height (cm)	178.02	8.62	166.18	7.76
Weight (kg)	72.90	12.43	55.96	8.65
BMI (kg/m^2^)	22.92	3.36	20.25	2.78

BMI, body mass index.

The distribution of BMI values according to genders is presented in Table 2. There was a significant difference between genders in terms of BMI values with higher BMI in boys (*P* < 0.001).

**Table 2.** Distribution of BMI groups between genders

BMI	Boys		Girls	
	*n*	%	*n*	%
Underweight	101	9.3	396	35.4
Normal	737	67.8	659	58.9
Overweight	206	19.0	56	5.0
Obese	43	4.0	8	0.7

BMI = body mass index.

The distribution of life style characteristics of the subjects according to gender and BMI groups is summarized in Table 3. With regard to smoking status, for boys and girls, 31.6–18.4% of the subjects were active smokers (*n* = 375–230), whereas 4.1–0.9% had quitted (*n* = 48–36). Smoking status of the participants was evaluated according to gender and BMI categories. There was a significant correlation between them (*P* < 0.001). Among male and female smokers, 83.4–87.8% were smoking a packet of cigarettes or lower in a day, whereas 17.6–12.2% were smoking more than a packet.

**Table 3.** Distribution of life style factors between genders and BMI groups

		Gender			BMI				
		Boys *n* (%)	Girls *n* (%)	*P*	Underweight *n* (%)	Normal *n* (%)	Over weight *n* (%)	Obese *n* (%)	*P*
Smoking status	Nonsmoker	762 (64.3)	985 (78.7)	**<0.001**	385 (77.8)	1005 (72.4)	158 (60.3)	33 (68.8)	**<0.001**
	Quitter	48 (4.1)	36 ( %.9)		13 (2.6)	49 (3.5)	15 (5.7)	-	
	Current smoker	375 (31.6)	230 (18.4)		97 (19.6)	334 (24.1)	89 (34)	15 (31.2)	
Daily number of cigarettes	15	72 (20.5)	59 (26.6)	**0.005**	30 (31.9)	62 (19.7)	24 (28.6)	2 (11.8)	0.068
	6–10	84 (23.9)	67 (30.2)		26 (27.7)	85 (27.1)	19 (22.6)	3 (17.6)	
	1120	134 (38.1)	69 (31.1)		25 (26.6)	120 (38.2)	30 (35.7)	6 (35.3)	
	⩾21	62 (17.6)	27 (12.2)		13 (13.8)	47 (15)	11 (13.1)	6 (35.3)	
Family history	No	713 (59.6)	702 (56.0)	0.069	313 (63.0)	828 (59.3)	131 (50)	21 (41.2)	**<0.001**
	Yes	483 (40.4)	552 (44.0)		184 (37.0)	568 (40.7)	131 (50.0)	30 (58.8)	
Fat consumption	Butter	264 (22.2)	155 (12.4)	**0.000**	91 (18.4)	241 (17.3)	49 (18.9)	11 (21.6)	**0.000**
	Margarine	186 (15.7)	120 (9.6)		73 (14.7)	173 (12.4)	28 (10.8)	7 (13.7)	
	Olive oil	632 (53.2)	786 (63)		305 (61.6)	798 (57.3)	138 (53.3)	33 (64.7)	
	Corn, soy or sunflower seed oil	397 (33.4)	426 (34.1)		152 (30.7)	481 (34.5)	92 (35.5)	16 (31.4)	
	Hazelnut oil	46 (3.9)	60 (4.8)		21 (4.2)	63 (4.5)	9 (3.5)	3 (5.9)	
	No fat consumption	18 (1.5)	17 (1.4)		8 (1.6)	16 (1.1)	5 (1.9)	3 (5.9)	

BMI = body mass index.

First-degree relatives (mothers, fathers, or siblings) of 40.4–44.0% (*n* = 483–552) of the subjects were under treatment for heart disease, diabetes, or hypertension. Participants’ family history of the disease was assessed according to BMI values, and there was a significant relationship between them (*P* < 0.001).

Regarding fat consumption routines for both boys and girls, it was determined that 22.2–12.4% (*n* = 264–155) of the subjects were using butter, 15.7–9.6% (*n* = 186–120) were using margarine, 53.2–63% (*n* = 632–786) were using olive oil, 33.4–34.1% (*n* = 397–426) were using sunflower seed/corn/soy oil, and 3.9–4.8% (*n* = 46–60) were using hazelnut oil. Fat consumption of the participants was evaluated according to gender and BMI categories and a significant relationship between them (*P* < 0.001).

With regard to the importance given by the subjects to CVD risk factors, the leading responses were as follows: high cholesterol (65.6%), stress (65.5%), hypertension (57.3%), smoking (56%), obesity(55.8%), diabetes (47.3%), inactivity (45.6%), alcohol consumption (40.7%), similar disease in family history (38.3%), unhealthy diet (35.7%), exposure to second-hand cigarette smoke (31.1%), and poor economic status (26.3%). Comparison between genders revealed that boys gave significantly lower importance to these risk factors compared with women. Comparison between BMI groups revealed that obese subjects gave significantly lower importance to these risk factors as well (Table 4).

**Table 4.** Perspectives of subjects about risk factors of cardiovascular diseases according to gender, BMI groups, family history of disease, and smoking status

	Not important, *n* (%)	Little important, *n* (%)	Moderately important, *n* (%)	Important, *n* (%)	Very important, *n* (%)	*P*
Unhealthy diet						
Male	204 (17.9)	204 (17.9)	247 (21.7)	373 (32.7)	111 (9.7)	<0.001
Female	97 (8.3)	178 (15.3)	263 (22.5)	451 (38.6)	178 (15.3)	
Fam.Hist.Disease (+)	97 (10)	158 (16.2)	211 (21.7)	367 (37.7)	141 (14.5)	0.0001
Fam.Hist.Disease (−)	204 (15.3)	224 (16.8)	299 (22.4)	457 (34.3)	148 (11.1)	
Smoker	87 (15.29)	105 (18.45)	125 (21.97)	195 (34.27)	57 (10.02)	0.043
Quitter	10 (12.35)	9 (11.11)	15 (18.52)	38 (46.91)	9 (11.11)	
Nonsmoker	204(12.42)	265 (16.14)	366 (22.29)	586 (35.69)	221 (13.46)	
Obesity						
Male	43 (3.7)	53 (4.5)	80 (6.8)	449 (38.2)	549 (46.8)	<0.001
Female	9 (0.7)	13 (1)	48 (3.9)	374 (30)	801 (64.3)	
Underweight	5 (1)	11 (2.2)	26 (5.3)	156 (31.5)	297 (60)	<0.001
Normal	29 (2.1)	42 (3.1)	71 (5.2)	456 (33.1)	778 (56.5)	
Overweight	6 (2.3)	10 (3.8)	18 (6.9)	101 (38.8)	125 (48.1)	
Obese	5 (9.8)	0 (0)	5 (9.8)	20 (39.2)	21 (41.2)	
Fam.Hist.Disease (+)	15 (1.5)	20 (2)	56 (5.5)	348 (34.2)	580 (56.9)	0.041
Fam.Hist.Disease (−)	37 (2.6)	46 (3.3)	72 (5.1)	475 (33.9)	770 (55)	
Smoker	24 (4.04)	20 (3.37)	32 (5.39)	227 (38.22)	291 (48.99)	0.000
Quitter	0 (0)	1 (1.19)	4 (4.76)	26 (30.95)	53 (63.1)	
Nonsmoker	27 (1.56)	43 (2.49)	92 (5.33)	567 (32.83)	998 (57.79)	
Inactivity						
Male	29 (2.5)	64 (5.5)	129 (11)	442 (37.7)	507 (43.3)	0.005
Female	23 (1.9)	42 (3.4)	132 (10.7)	448 (36.2)	591 (47.8)	
Underweight	8 (1.6)	24 (4.9)	58 (11.8)	185 (37.8)	215 (43.9)	0.002
Normal	30 (2.2)	65 (4.7)	140 (10.2)	503 (36.6)	635 (46.2)	
Overweight	4 (1.6)	7 (2.7)	32 (12.5)	99 (38.5)	115 (44.7)	
Obese	4 (7.8)	0 (0)	7 (13.7)	23 (45.1)	17 (33.3)	
Poor economic status						
Male	127 (10.9)	242 (20.7)	251 (21.5)	285 (24.4)	264 (22.6)	0.027
Female	102 (8.3)	213 (17.4)	291 (23.8)	344 (28.1)	273 (22.3)	
Underweight	35 (7.1)	84 (17.1)	114 (23.3)	144 (29.4)	113 (23.1)	0.001
Normal	133 (9.8)	258 (19)	319 (23.4)	347 (25.5)	304 (22.3)	
Overweight	32 (12.5)	51 (19.8)	61 (23.7)	63 (24.5)	50 (19.5)	
Obese	7 (13.7)	15 (29.4)	12 (23.5)	8 (15.7)	9 (17.6)	
Smoker	72 (12.29)	102 (17.41)	117 (19.97)	150 (25.6)	145 (24.74)	0.027
Quitter	8 (9.76)	12 (14.63)	22 (26.83)	20 (24.39)	20 (24.39)	
Nonsmoker	145 (8.48)	339 (19.82)	400 (23.39)	456 (26.67)	370 (21.64)	
Stress						
Male	24 (2.1)	21 (1.8)	106 (9.1)	327 (28.2)	682 (58.8)	<0.001
Female	8 (0.6)	20 (1.6)	49 (4)	272 (22)	888 (71.8)	
Underweight	3 (0.6)	5 (1)	24 (4.9)	123 (25)	337 (68.5)	<0.001
Normal	22 (1.6)	25 (1.8)	89 (6.5)	346 (25.4)	881 (64.6)	
Overweight	5 (2)	6 (2.3)	25 (9.8)	64 (25)	156 (60.9)	
Obese	1 (2)	4 (7.8)	4 (7.8)	13 (25.5)	29 (56.9)	
Similar disease in family history						
Male	58 (5)	101 (8.7)	220 (18.9)	417 (35.7)	371 (31.8)	<0.001
Female	20 (1.6)	55 (4.5)	152 (12.3)	457 (37.1)	547 (44.4)	
Underweight	10 (2)	26 (5.3)	77 (15.7)	163 (33.3)	214 (43.7)	0.005
Normal	44 (3.2)	100 (7.3)	202 (14.8)	510 (37.3)	513 (37.5)	
Overweight	8 (3.1)	20 (7.8)	44 (17.2)	94 (36.7)	90 (35.2)	
Obese	5 (10.2)	1 (2)	6 (12.2)	22 (44.9)	15 (30.6)	
Smoking						
Male	81 (6.9)	54 (4.6)	118 (10.1)	295 (25.2)	621 (53.1)	<0.001
Female	51 (4.2)	47 (3.8)	87 (7.1)	321 (26.2)	718 (58.7)	
Underweight	17 (3.5)	26 (5.3)	38 (7.7)	139 (28.3)	271 (55.2)	0.024
Normal	75 (5.5)	60 (4.4)	113 (8.3)	346 (25.4)	769 (56.4)	
Overweight	21 (8.2)	4 (1.6)	29 (11.3)	62 (24.1)	141 (54.9)	
Obese	6 (12)	3 (6)	8 (16)	10 (20)	23 (46)	
Smoker	39 (6.61)	39 (6.61)	79 (13.39)	170 (28.81)	263 (44.58)	<0.001
Quitter	3 (3.66)	7 (8.54)	7 (8.54)	23 (28.05)	42 (51.22)	
Nonsmoker	88 (5.16)	55 (3.22)	118 (6.91)	418 (24.49)	1028 (60.22)	
Hypertension						
Male	36 (3.1)	28 (2.4)	125 (10.7)	394 (33.8)	582 (50)	<0.001
Female	16 (1.3)	22 (1.8)	73 (5.9)	329 (26.7)	790 (64.2)	
Underweight	5 (1)	6 (1.2)	40 (8.2)	145 (29.8)	291 (59.8)	0.017
Normal	33 (2.4)	33 (2.4)	118 (8.6)	404 (29.6)	778 (57)	
Overweight	6 (2.3)	8 (3.1)	21 (8.1)	90 (34.9)	133 (51.6)	
Obese	2 (4.1)	0 (0)	4 (8.2)	17 (34.7)	26 (53.1)	
Smoker	22 (3.72)	15 (2.54)	60 (10.15)	162 (27.41)	332 (56.18)	0.026
Quitter	1 (1.22)	2 (2.44)	3 (3.66)	27 (32.93)	49 (59.76)	
Nonsmoker	29 (1.7)	33 (1.93)	135 (7.9)	527 (30.84)	985 (57.64)	
Diabetes mellitus						
Male	50 (4.3)	58 (5)	150 (13)	416 (36)	482 (41.7)	<0.001
Female	27 (2.2)	55 (4.5)	126 (10.3)	369 (30.2)	643 (52.7)	
High cholesterol level						
Male	24 (2.1)	29 (2.5)	86 (7.4)	344 (29.7)	676 (58.3)	<0.001
Female	11 (0.9)	12 (1)	50 (4.1)	262 (21.5)	881 (72.5)	
Underweight	2 (0.4)	7 (1.4)	26 (5.4)	117 (24.2)	331 (68.5)	0.001
Normal	23 (1.7)	23 (1.7)	79 (5.8)	337 (24.9)	891 (65.9)	
Overweight	7 (2.7)	7 (2.7)	14 (5.4)	85 (32.8)	146 (56.4)	
Obese	2 (4)	0 (0)	3 (6)	15 (30)	30 (60)	
Smoker	18 (3.08)	16 (2.74)	40 (6.84)	145 (24.79)	366 (62.56)	0.003
Quitter	0 (0)	1 (1.28)	5 (6.41)	17 (21.79)	55 (70.51)	
Nonsmoker	17 (1)	24 (1.41)	91 (5.36)	440 (25.9)	1127 (66.33)	
Exposure to second-hand cigarette smoke						
Male	99 (8.5)	163 (14)	264 (22.6)	357 (30.6)	285 (24.4)	0.001
Female	50 (4.1)	133 (10.8)	240 (19.4)	391 (31.7)	420 (34)	
Underweight	17 (3.5)	57 (11.6)	115 (23.5)	143 (29.2)	158 (32.2)	0.039
Normal	91 (6.7)	174 (12.7)	275 (20.1)	435 (31.8)	392 (28.7)	
Overweight	19 (7.3)	32 (12.3)	59 (22.7)	82 (31.5)	68 (26.2)	
Obese	5 (10)	4 (8)	12 (24)	14 (28)	15 (30)	
Smoker	82 (13.95)	92 (15.65)	132 (22.45)	169 (28.74)	113 (19.22)	0.000
Quitter	2 (2.41)	9 (10.84)	23 (27.71)	29 (34.94)	20 (24.1)	
Nonsmoker	63 (3.67)	194 (11.3)	345 (20.09)	546 (31.8)	569 (33.14)	

BMI = body mass index.

Fam.Hist.Disease, cardiovascular disease in family history.

CVD risk factors were evaluated according to gender and BMI status. In terms of obesity, a significant relationship was detected between diet, gender, family disease status, and CVD. Less physical activity, stress, and having similar disease in the family were significantly related with gender and BMI. While passive smoking, alcohol consumption, smoking, and hypertension were related with only BMI, only diabetes was significantly related with gender (Table 4).

The question of ‘preventive measures that risky subjects should take’ was considered significantly more important by women than men (Table 5). Smoking, alcohol consumption, and stress withdrawal were significantly different across BMI groups, and these factors seemed to be less important in obese groups compared with other groups (Table 5).

**Table 5.** Preventive measures for the subjects at risk of cardiovascular disease according to gender, BMI groups, family history of disease, and smoking status

	Totally disagree, *n* (%)	Disagree, *n* (%)	Unsure, *n* (%)	Agree, *n* (%)	Totally agree, *n* (%)	*P*
Should not smoke						
Male	26 (2.2)	7 (0.6)	25 (2.1)	137 (11.7)	977 (83.4)	<0.001
Female	8 (0.7)	5 (0.4)	16 (1.3)	120 (9.8)	1080 (87.9)	
Underweight	1 (0.2)	1 (0.2)	10 (2)	50 (10.2)	430 (87.4)	0.037
Normal	22 (1.6)	8 (0.6)	25 (1.8)	144 (10.5)	1168 (85.4)	
Overweight	6 (2.3)	1 (0.4)	2 (0.8)	25 (9.6)	227 (87)	
Obese	1 (2)	1 (2)	1 (2)	8 (16)	39 (78)	
Smoker	17 (2.9)	5 (0.9)	17 (2.9)	99 (16.9)	448 (76.5)	0.000
Quitter	1 (1.2)	1 (1.2)	3 (3.7)	11 (13.4)	66 (80.5)	
Nonsmoker	15 (0.9)	6 (0.4)	21 (1.2)	146 (8.5)	1531 (89.1)	
Should not eat fat						
Male	17 (1.5)	20 (1.7)	67 (5.7)	328 (28.1)	735 (63)	<0.001
Female	8 (0.6)	4 (0.3)	22 (1.8)	225 (18.2)	976 (79)	
Should avoid stress						
Male	12 (1)	10 (0.9)	40 (3.4)	293 (25.2)	810 (69.5)	<0.001
Female	5 (0.4)	3 (0.2)	17 (1.4)	227 (18.4)	982 (79.6)	
Underweight	1 (0.2)	0 (0)	7 (1.4)	101 (20.6)	381 (77.8)	0.001
Normal	12 (0.9)	10 (0.7)	32 (2.3)	297 (21.7)	1017 (74.3)	
Overweight	3 (1.2)	1 (0.4)	10 (3.8)	64 (24.6)	182 (70)	
Obese	0 (0)	1 (2)	5 (10)	8 (16)	36 (72)	
Smoker	8 (1.4)	5 (0.9)	24 (4.1)	125 (21.3)	426 (72.4)	0.019
Quitter	1 (1.2)	1 (1.2)	1 (1.2)	19 (23.2)	60 (73.2)	
Nonsmoker	8 (0.5)	7 (0.4)	32 (1.9)	375 (21.9)	1293 (75.4)	
Should do exercise						
Male	19 (1.6)	16 (1.4)	46 (3.9)	273 (23.4)	815 (69.7)	0.003
Female	7 (0.6)	7 (0.6)	41 (3.3)	279 (22.6)	900 (72.9)	
Should not consume alcohol						
Male	24 (2.1)	21 (1.8)	106 (9.1)	327 (28.2)	682 (58.8)	<0.001
Female	8 (0.6)	20 (1.6)	49 (4)	272 (22)	888 (71.8)	
Underweight	7 (1.4)	9 (1.8)	37 (7.5)	109 (22.2)	329 (67)	0.004
Normal	43 (3.2)	41 (3)	108 (7.9)	274 (20.1)	898 (65.8)	
Overweight	12 (4.6)	10 (3.8)	24 (9.2)	51 (19.6)	163 (62.7)	
Obese	3 (6)	0 (0)	7 (14)	10 (20)	30 (60)	
Smoker	31 (5.3)	27 (4.6)	65 (11.1)	131 (22.4)	331 (56.6)	0.000
Quitter	2 (2.5)	2 (2.5)	8 (9.9)	13 (16.1)	56 (69.1)	
Nonsmoker	38 (2.2)	37 (2.2)	119 (6.9)	345 (20.1)	1175 (68.6)	
Should not become obese						
Male	19 (1.6)	24 (2.1)	90 (7.7)	384 (33)	647 (55.6)	<0.001
Female	5 (0.4)	10 (0.8)	54 (4.4)	373 (30.4)	784 (63.9)	
Should check blood pressure						
Male	22 (1.9)	19 (1.6)	85 (7.3)	360 (30.9)	679 (58.3)	<0.001
Female	6 (0.5)	6 (0.5)	42 (3.4)	329 (26.9)	841 (68.7)	
Fam.Hist.Disease (+)	7 (0.7)	8 (0.8)	45 (4.5)	277 (27.5)	671 (66.6)	0.031
Fam.Hist.Disease (–)	21 (1.5)	17 (1.2)	82 (5.9)	412 (29.8)	849 (61.5)	
Should avoid exposing to smoke						
Male	38 (3.3)	44 (3.8)	122 (10.5)	365 (31.4)	594 (51.1)	<0.001
Female	15 (1.2)	24 (2)	116 (9.5)	385 (31.4)	685 (55.9)	
Fam.Hist.Disease (+)	13 (1.3)	36 (3.6)	91 (9)	314 (31.2)	554 (55)	0.015
Fam.Hist.Disease (–)	40 (2.9)	32 (2.3)	147 (10.7)	436 (31.6)	725 (52.5)	
Smoker	26 (4.5)	32 (5.5)	69 (11.9)	187 (32.2)	266 (45.9)	0.000
Quitter	2 (2.5)	1 (1.2)	10 (12.4)	27 (33.3)	41 (50.6)	
Nonsmoker	22 (1.3)	35 (2)	159 (9.3)	532 (31.1)	965 (56.3)	

BMI = body mass index.

Fam.Hist.Disease, cardiovascular disease in family history.

## Discussion

In a previous cross-sectional study in the same population, we investigated the relation between eating habits and a high BMI among freshman students (Gunes *et al*., 2012). Our observations indicated that cultural and local issues guide the adoption of healthier feeding behaviors among university students. To extend our analysis further, we analyzed the awareness of CVD risk factors among university students in relation to the four categories of BMI in the same cross-sectional study. Among our participants, 322 were overweight or obese (BMI 25). The Turkey Health Interview Survey, conducted by TUIK (2016), found that (for the 15+ age group) 38.6% of men were overweight and 15.2% obese, while 30.1% of women were overweight and 23.9% obese (TUİK; http://tuik.gov.tr/PreTablo.do?alt_id=1095). Similarly, male gender was an independent predictor of obesity/overweight status compared with female gender in this study.

The present study was performed on the first-year university students to determine the awareness of young people about risk factors of CVD, such as obesity, unhealthy nutrition, or lifestyle behaviors. These behaviors are very important since at least 80% of CVD most commonly occur due to conventional risk factors. Prevention of these risk factors might decrease morbidity and mortality by 80–90% (Çengel, 2010). The presence of unfavorable lifestyle behaviors is responsible for the majority of deaths due to CVD (Mokdad *et al*., 2004).

The awareness of healthy lifestyle behavior is crucial, particularly in young age, since the structural basis of CVD, such as atherosclerotic plaque, prepares their foundation in early years. Being aware of healthy living in early ages would help to take cardioprotective precautions before the occurrence of irreversible changes in the organism (Hayman *et al*., 2004).

Obesity, a considerable risk factor for CVD, is a common disorder that may be considered a strong stimulant to increase the level of awareness in the population. In the present study population, it was observed that awareness about CVD risk factors was lower in the obese group compared with the normal-weight group. Reiner *et al*. (2012) have found that the perception and knowledge about risk factors of CVD were insufficient among young people. In another study, Vanhecke *et al*. (2006) have investigated the awareness, knowledge, and perception of adolescents about heart diseases and found that young people generally had similar levels of awareness compared with their counterparts. In this the healthy population, because of some of them that having a family member with CVD, their awareness of healthy living are expected to raise; however, Hunt *et al*. (1986) showed that this is not the determinant of healthy lifestyle behaviors in young adults. On the contrary, having a family history of CVD is a strong predictor of subsequent disease if not taken under control at the right time, and healthy lifestyle should be established among young people regardless of the risk of parental inheritance. However, studies on college students have revealed that students generally adopted their parents’ behaviors and habits that contributed to CVD (Tamragouri *et al*., 1986).

Gender is another important factor in the awareness of CVD risk factors. In literature, women presented more awareness about risk factors of CVD (Mosca *et al*., 2004), whereas men have the opinion that their health status is better compared to that of women (Romero, 2004). Women are also under a high risk of asymptomatic CVD. A study by Çengel (2010) has revealed that cases of a structural heart disease proceeding to a sudden cardiac death are lower in women than men, and that the identification of risk factors is a major step in protection from CVD, especially in women who have asymptomatic cardiovascular disease.

Christian *et al*. (2007) determined that the level of awareness among American females about CVD risk factors increased by twofold in the last decade. Studies have demonstrated that awareness about the symptoms of heart diseases is increasing among women than men (Zbierajewski-Eischeid and Loeb, 2009). Consistent with literature, the results of the present study revealed that the level of awareness about CVD risk factors is higher among women.

The risk factors of mortality in CVD are generally similar across studies, which are obesity, smoking, and high cholesterol levels (Anding *et al*., 1996; Navas-Nacher *et al*., 2001). The awareness about these factors is generally similar as well. However, Steptoe *et al*. studied the trends of healthy lifestyle factors among university students from 13 European countries (Belgium, England, France, Germany, Greece, Hungary, Iceland, Ireland, Italy, The Netherlands, Poland, Portugal, and Spain) between 1990 and 2000 and revealed that there is a downward trend in the beliefs about the importance of diet and quitting smoking. Moreover, the study also indicated that there was an increasing trend in smoking during this period (Steptoe *et al*., 2002; Vanhecke *et al*., 2006). According to TURDEP II (Satman *et al*., 2011) study results, in the Turkish society, the proportion of smokers has decreased. In the general population, smoking rates decreased from 29.8% in 1998 to 17.3% in 2010. However, male smokers are over 30%. The rate of smoking cessation increased from 3.8% to 12.1%. These results show that during the 12-year period, the proportion of smokers in Turkey decreased by 42% (Satman *et al*., 2011).

In the present study, cigarette smoking was associated with increased awareness of risk factors of CVD. This result is in agreement with a previous finding that smoking decreases healthy lifestyle factors, and it is a major independent risk factor of CVD (Talukder *et al*., 2011).

Another important protective factor against the development of CVD is maintaining healthy eating behaviors. Studies have shown that the Mediterranean diet, which is rich in fruits, whole grains, fish, vegetables, and especially olive oil, has beneficial effects on cardiovascular protection (Estruch *et al*., 2006). The American Heart Association has generalized their recommendations for total calorie intake and healthy eating behaviors for children and adolescents (Gidding *et al*., 2005; Steinberger *et al*., 2009; Sarpong *et al*., 2017). In the present study, the role of diet in CVD was also questioned, and it was found that subjects with both a family member with CVD and who are nonsmokers are more aware of the importance of dietary factors in the development of CVD. This awareness could be accounted for the awareness of healthy lifestyle factors in this population.

Having a relatively large sample size, including healthy subjects from a wide range of socio-economical groups, is a strength of this epidemiological study. This large sample belonging to Turkish universities indicates that the subjects represented the target population suitably. And, there has been no comprehensive study in Turkey evaluating the perception and awareness of young people about the risks of CVD.

Followings are the potential limitations: food quantity was not measured, self-reported BMI was used, body fat content was not measured, and physical activity was not questioned.

The results of the present study were generally consistent with literature data. Previous studies on this topic have also reported that young people have rather limited awareness of certain risk factors of CVD, and suggested that strategies should be developed to enhance the level of knowledge about healthy lifestyle behaviors in general population (Dodani *et al*., 2004; Aminde *et al*., 2017).

## Conclusion

Primary prevention is vital to combat the consequences of CVD in developing countries where resources are limited. The present study showed that awareness of CVD needs to improve significantly among the university students. Male gender and obesity are important determinants of CVD awareness. It is crucial that conscious-raising interventions are needed for risk groups (**ie**, obese, smoker, family history). School and community programs (**ie**, SMART Project, Bogalusa Heart Study) prove to be efficient interventions that can lead similar efforts in developing countries. Universities should include awareness programs on CVD in their curriculum. It has been emphasized that primary healthcare workers are very important in the screening of CVD risk factors in an opportunistic and systematic way and in providing consultancy on changing risky behaviors (diet, smoking, etc.). Therefore, it is of utmost importance that primary healthcare workers make interventions to reduce the risk level by determining the CVD risk.
