# Awareness of cardiovascular risk factors among university students in Turkey – CORRIGENDUM

**DOI:** 10.1017/S1463423619000823

**Published:** 2019-11-08

**Authors:** F. Esra Güneş, Nural Bekiroglu, Neşe Imeryuz, Mehmet Agirbasli

In the above article incorrect author affiliations were submitted to Primary Health Care Research & Development.

F. Esra Güneş’ affiliation is the previously omitted Department of Nutrition and Dietetic, Faculty of Health Science, Marmara University, İstanbul, Turkey. This has become the new affiliation 1, while the the previous affiliations 1 – 3 have been re-numbered as 2 – 4.

The original article has now been updated to reflect this.
